# Investigation of single nucleotide polymorphisms based on the intronic sequences of the propylene alcohol dehydrogenase gene in Chinese tobacco genotypes

**DOI:** 10.1080/13102818.2014.907651

**Published:** 2014-07-03

**Authors:** Ji-Cheng Wei, En-Jian Qiu, Hui-Yan Guo, Ai-Ping Hao, Rong-Ping Chen

**Affiliations:** ^a^ Department of Biology, Mudanjiang Teachers College, Mudanjiang, P.R. China; ^b^ Laboratory of Breeding, Mudanjiang Tobacco Research Institute, Mudanjiang, P.R. China

**Keywords:** tobacco, propylene alcohol dehydrogenase gene, intron polymorphisms

## Abstract

A pair of primers was designed to amplify the propylene alcohol dehydrogenase gene sequence based on the cDNA sequence of the tobacco allyl-alcohol dehydrogenase gene. All introns were sequenced using traditional polymerase chain reaction (PCR) methods and T-A cloning. The sequences from common tobacco (*Nicotiana tabaccum* L.) and rustica tobacco (*Nicotiana rustica* L.) were analysed between the third intron and the fourth intron of the propylene alcohol dehydrogenase gene. The results showed that the alcohol dehydrogenase gene is a low-copy nuclear gene. The intron sequences have a combination of single nucleotide polymorphisms and length polymorphisms between common tobacco and rustica tobacco, which are suitable to identify the different germplasms. Furthermore, there are some single nucleotide polymorphism sites in the target sequence within common tobacco that can be used to distinguish intraspecific varieties.

## Introduction

Tobacco (*Nicotiana* sp.) is one of many plants currently used as models for molecular studies and is an economically important crop. The interspecific phylogenetic relationships within tobacco are complicated due to complex hybridization events within the tobacco lineage. DNA molecular markers have been used to identify germplasms and to accelerate the breeding cycle. The second generation of molecular markers that include random amplification of polymorphic DNA (RAPD), amplified fragment length polymorphism (AFLP) and simple sequence repeats (SSR) has been employed broadly to differentiate tobacco varieties,[1–4] while a third generation of molecular markers, intron polymorphisms (IPs) based on single nucleotide polymorphisms (SNPs), has been used as well. Abundant noncoding sequences, introns, are interspersed in the eukaryote genome and have been shown to accumulate considerable nucleotide variations.[5] At least half of the introns (>57,000) in tobacco are suitable for the exploitation of IP markers.[6] In this work, the regional IPs of the tobacco propylene alcohol dehydrogenase gene were analysed interspecifically and intraspecifically to assist in the identification of the germplasms studied.

## Materials and methods

The tobacco samples used were all provided by the Mudanjiang Tobacco Research Institute (Table 1). The genomic DNA was isolated from tobacco samples, using a plant genomic DNA extraction kit (TianGen Bio Inc., China). With a template from common tobacco named C1, a pair of primers was designed according to the mRNA for the tobacco propylene alcohol dehydrogenase gene (GenBank: AB036735.1). The two primers were designed to amplify the DNA for tobacco propylene alcohol dehydrogenase gene, and the sequences were as follows, C1-S: 5′-CAAAAGTTAAGAAAATGGC-3′, C1-A: 5′-AGTCCAGTGGCTTAACTC-3′. The PCR materials included 5 μL of 10 × PCR buffer, 4 μL of dNTP (2.5 mmol/L each), 1 μL of each primer (10 μmol/L), 1 μL of DNA template and 1.5 U of ex-Taq DNA polymerase (Takara Bio Inc., Tokyo, Japan). PCR was performed at 94 °C for 5 min, then 94 °C for 30 s, 58 °C for 30 s, 72 °C for 1 min for 30 cycles, with a hold at 72 °C for 20 min. The PCR products were sequenced after their detection on agarose/EB gels. The intron distribution was analysed according to the GT-AG rule in the target gene with reference to its cDNA sequence.

**Table 1.** Tobacco samples used in this study.

No.	Varieties	Sample type	No.	Varieties	Sample type
S1	Yuqingheikouyan	Sun-shined tobacco	F6	9826-412C	Flue-cured tobacco
S2	Dejiangdajiwei	Sun-shined tobacco	F7	NC95	Flue-cured tobacco
S3	Huizeleyedabianyan	Sun-shined tobacco	F8	Longjiang851	Flue-cured tobacco
S4	Anmashanshaiyan-6	Sun-shined tobacco	F9	K326	Flue-cured tobacco
S5	Xuanchengliuyeyan	Sun-shined tobacco	F10	Delhi76	Flue-cured tobacco
S6	Shandongyan	Sun-shined tobacco	F11	RG11	Flue-cured tobacco
S7	Xiangyuntuyan-1	Sun-shined tobacco	R1	Mianyaxianglanhuayan	Rustica tobacco
S8	Daliuyetuyan	Sun-shined tobacco	R2	Xianfengjiayan	Rustica tobacco
S9	Mengbanshaiyan	Sun-shined tobacco	R3	Songbailanhuayan	Rustica tobacco
S10	Yongshengbianyan	Sun-shined tobacco	R4	Linxianxiaoyeyan	Rustica tobacco
S11	Nanpingshaiyan	Sun-shined tobacco	R5	Jinglexiaoyeyan	Rustica tobacco
S12	Husaxiaoliuye	Sun-shined tobacco	R6	Jiaoyixiaolanhua	Rustica tobacco
S13	Badahetuyan	Sun-shined tobacco	R7	Jiaochengxiaolanhua	Rustica tobacco
S14	Puzelingshaiyan	Sun-shined tobacco	R8	Pianguanxiaolanhua	Rustica tobacco
F1	9861-81	Flue-cured tobacco	R9	Hequxiaoyanye	Rustica tobacco
F2	Longjiang925	Flue-cured tobacco	R10	Wuzhaixiaolanhua	Rustica tobacco
F3	9826-412C	Flue-cured tobacco	R11	Hunyuanxiaolanhua	Rustica tobacco
F4	9861-81	Flue-cured tobacco	R12	Pingwulanhua	Rustica tobacco
F5	9826-412C	Flue-cured tobacco	C1	Beinhart1000-1	Cigar

The specific primers were designed to target the boundary between the third intron and the fourth intron of the tobacco propylene alcohol dehydrogenase gene, and the IP sequences were identified as follows, IP-S: GTGCTGGAAGCAAAGAAAAG, IP-A: CAATTCCATCAGGGAAGTAC. The PCR materials included 5 μL of 10 × PCR buffer, 4 μL of dNTP (2.5 mmol/L each), 1 μL of each primer (10 μmol/L), 1 μL of the DNA template and 1.5 U of *r*Taq DNA polymerase (Takara Bio Inc., Tokyo, Japan).

PCR was performed at 94 °C for 5 min, then 94 °C for 30 s, 58 °C for 30 s, 72 °C for 1 min for 30 cycles, with a hold at 72 °C for 20 min. The PCR products were ligated to a pGEM-T vector (Promega Corporation, Madison, WI, USA) and sequenced on an ABI 3730 sequencer after electrophoretic detection in a 1.0% agarose/EB gel.

## Results and discussion

The propylene alcohol dehydrogenase gene of sample C1 contained four introns and five exons that were 225, 178, 167, 83 and 379 bp long (Online Supplemental Appendix 1). The boundary sequence between the intron and exon segments were the sites of interest for which a primer was designed to target.

**Figure 1.**
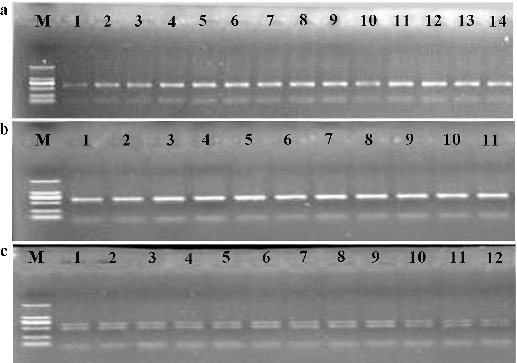
Agarose/EB gel electrophoresis results of the amplification of the target region in sun-shined tobacco samples (a); flue-cured tobacco samples (b); and rustica tobacco samples (c).

The target PCR product from the rustica tobacco (*Nicotiana rustica*) exhibited two bands when both primers, IP-S and IP-A, were used, while those of common tobacco (sun-shined tobacco and flue-cured tobacco) exhibited only one band on the 1.0% agarose/EB gel (Figure 1). The results showed that these primers have the ability to differentiate common tobacco from rustica tobacco, and that there are two members of the propylene alcohol dehydrogenase gene with length polymorphisms in rustica tobacco.

The sequence results also displayed two copies of propylene alcohol dehydrogenase gene with length polymorphisms in common tobacco in a 3.0% agarose/EB gel, as two bands of the PCR product were able to be differentiated from each other (data not shown).

In the common tobacco, the shorter fragment was 559-bp long in the target region, and the two introns found were 223 and 253 bp. There were no length differences in this fragment among the 12 samples sequenced, and the consensus sequence was as follows:

GTAAGTTCCATTGCGTTGGATCTTCATAGTGTTGAATCTATCTTCATTCTTCGTTATGTTAATCATTGTGCTCCGTCCACCATCAAACCCAAAGGTTGGGGTTGGGGTTGGGGTTAGGGTTGGGTGGGTTATGCCTTTGGGGGGAAAGAGAGGTTTTAGTTTAATAGACCATGATGTTTAAAACTCTTGTTATTTAATGTCTCTTTTTTATTCATTTCCAAAGGTTGATCTGTTGAAGAGCAAATTTGGTTTTGACGAGGCTTTTAACTATAAAGAGGAGCAGGATTTAAATGCAGCTTTGAAGAGGTGAGTAATCTTACTTCCTGCTACAAAACAATCTACCTATTGTACAAAAGCAGTTAAAGATGTTCTGTGAAAAGTATGATGATATTATGTAGATAGTGTGAAACTTATAGAAAGACTTTAAGATATGCCTACGAAAACCGAGGAACATGGATCATTTCAAATCTGGCTTTTCATGAATATGGAACCCCATTCACTAGATTTTAGTGCGAAATTCCTAGACTTGAAGTGACTGGAAATACTGGTTTGTTATTAG

In the common tobacco, the longer sequence obtained was between 588 and 591-bp long in the target region, and the two introns were either between 194 and 197 bp or 311 bp in length. The sequences from 13 samples were analysed with the software ClusterW2 (www.ebi.ac.uk/Tools/clustalw2/). The results indicated that there were two transitions and one transversion in addition to three gaps within the third intron. The two transitions and one transversion were also present within the fourth exon, but no amino acid replacement had occurred. There were four transitions and one transversion within the fourth intron as well (data shown in Online Supplemental Appendix 2). These polymorphic sites within the third and fourth intron may function as references to distinguish the varieties of common tobacco studied here.

In the rustica tobacco, the shorter sequences were 442-bp long in the target region, and the two introns found were 195 and 164 bp in length. No length polymorphisms were found in this fragment among the 12 samples sequenced, and the consensus sequence obtained was as follows:

GTAAGTTCCTTCATAGTGTTGAAGCTCTCTTCATTCTTCGTTATGTTAATTATTGTGCTCCGTCCTCCATCAAACCCAAAGGTTGGGGTTTTGTGGGTTATGAGGAGGAGAAAGGGGTATACCTTTGGGGGGGAAGAGATGTTTTAGTTTAATAGACCATGATGTTTAACTCTCTTTTTATATTCATTTCCAAAGGTTGATCTGTTGAGGAACAAATTTGGTTTTGACGAAGCTTTTAACTATAAAGAGGAGCAGGACTTAAATGCAGCTTTGAAGAGGTGAGTAATCTTACTACTTGGCCTGGTGTTAGAGAAGCAGTTAGCAACAGCTTTTCATCAATATTTCCATAGAATGATATTTATAATGGAAGTCCCAAAGTAAAGATGTTCAGTGAAAAGTATGCCTGTGAAAACTGAGGGAAATAATACTGGTTTCTTATTAG

In the rustica tobacco, the longer sequence was 583-bp long in the target region, and the two introns found were 220 and 280 bp in length. No polymorphisms were found in this fragment among the 12 samples sequenced as well, and the consensus sequence was as follows:

GTAAGTTCCATTGCGTTGGATGTTCATAGTGTTGAAGCTATCTTCATTCTTCGTTATGTTAATCATTGTGTTCTGTCCACCGTCAAACCCAAAGGTTGGGGTTTTGTGGGTTATGAGGAGGAGAAAGGTATACCTTTGGGGGGAAGAGATGTTTTAGTTTAATAGACCATGATGTTTAACTATCTTTTTATTTAATGTCTCTTTTTATTCATTTCCAAAGGTTGATCTGTTGAAGAGCAAATTTGGTTTTGACGAAGCTTTTAACTATAAAGAGGAGCAGGACTTAAATTCAGCTTTGAAGAGGTGAGTAATCTTACTTCCTGTTAGAAAACAATCTACCTATTGTAACAAACTTGCCCTGGTGTTAGAGAAGCAGTTAGCAACAGCTTTTCCTTTTCATGTAAACGGAATCATCGATATTTCCATAGAATGATATTTATTCCTTGAACTGAAAAATAGAAGTCCCAAAGTAAAGATGTACAGTGAAAAGTATGAAGATTGTGTGAAAATTATAGAAAAGACTTTAGGATATGCGTATGAAAACTGAGGGAAATTCCTCCCTGGAATACTGGTTTCTTATTAG

In this study, the tobacco propylene alcohol dehydrogenase gene proved to be a low-copy nuclear gene. It could have formed as a result of divergent evolution after gene duplication, or by a parallel evolutionary process between the two sets of genomes, as the tobacco plants tested were all allotetraploid. Although the PCR product for the allyl-alcohol dehydrogenase gene of sample C1 was sequenced successfully, this does not mean that only one copy of the target gene is present in common tobacco. There were obvious intron length polymorphisms between common tobacco and rustica tobacco, which were mostly due to different modes of evolution of the two kinds of tobacco.

It is thought that the maternal ancestor of common tobacco was *Nicotiana sylvestris.*[7–9] It is also thought that its paternal ancestor was derived from either *N. tomentosiformis*,[10–15] *N. otophora* or from introgressive hybridization of *N. otophora* and *N. tomentosiformis* within the subgenus of common tobacco.[16,17] It has been shown that *N. rustica* originated from a natural chromosome duplication event after ancestral hybridization of *N. paniculata* and *N. undulate*.[18,19] Yang et al. [20] divided 48 tobacco samples into two categories, the *N. rustica* L. group and *Nicotiana tabaccum* L. group, after an analysis that used AFLP methods coupled with unweighted pair group method with arithmetic mean analysis, which was consistent with our results in this work. Furthermore, the two categories of tobacco can be distinguished using only one pair of primers according to the results of this work.

## Conclusions

The two copies of the propylene alcohol dehydrogenase gene found in tobacco each contained four introns and five exons. The length polymorphisms of the intron existed intraspecifically as well as interspecifically. The primers produced here, IP-S and IP-A, were able to easily differentiate common tobacco from rustica tobacco in a 1% agarose gel. Some SNPs of the intron within common tobacco were found, and may be useful in the identification of varieties of this species.

## Supplemental data

Supplemental data and research materials for this article can be accessed at http://dx.doi.org/10.1080/13102818.2014.907651.
